# Novel base triples in RNA structures revealed by graph theoretical searching methods

**DOI:** 10.1186/1471-2105-12-S13-S2

**Published:** 2011-11-30

**Authors:** Mohd Firdaus-Raih, Anne-Marie Harrison, Peter Willett, Peter J Artymiuk

**Affiliations:** 1 Department of Molecular Biology and Biotechnology, University of Sheffield, Sheffield S10 2TN, UK; 2 Department of Information Studies, University of Sheffield, Sheffield S10 2TN, UK; 3 School of Biosciences and Biotechnology, Faculty of Science and Technology, Universiti Kebangsaan Malaysia, 43600 UKM Bangi, Malaysia; 4 Present address: Biomedical Research Centre, Sheffield Hallam University, Sheffield S1 1WB, UK

## Abstract

**Background:**

Highly hydrogen bonded base interactions play a major part in stabilizing the tertiary structures of complex RNA molecules, such as transfer-RNAs, ribozymes and ribosomal RNAs.

**Results:**

We describe the graph theoretical identification and searching of highly hydrogen bonded base triples, where each base is involved in at least two hydrogen bonds with the other bases. Our approach correlates theoretically predicted base triples with literature-based compilations and other actual occurrences in crystal structures. The use of ‘fuzzy’ search tolerances has enabled us to discover a number of triple interaction types that have not been previously recorded nor predicted theoretically.

**Conclusions:**

Comparative analyses of different ribosomal RNA structures reveal several conserved base triple motifs in 50S rRNA structures, indicating an important role in structural stabilization and ultimately RNA function.

## Background

It is now clear that three-dimensional (3D) structure is as fundamental to the functionality of complex RNA molecules as it is for proteins. Awareness of the extent of the complexity and the diversity of RNA tertiary structures has expanded due to the availability of high resolution structures of large assemblies such as both ribosomal subunits [1-3], ribozymes [4,5], and the P4-P6 domain of the group I intron [6], in addition to the early transfer-RNA structures [7,8]. Base triples can provide key interactions in assembling a tertiary structure by docking a base-pair in a helical region, which may be either Watson-Crick or non-canonical, to a single-stranded nucleotide distant in the polynucleotide chain [9,10]. In addition, neighboring base triples can form stacks or triple helices: the first such example in the RNA shallow groove was observed in the crystal structure of a frame-shifting pseudoknot [11]. The occurrence of four base triples stacked together has also been observed in the structure of the *Tetrahymena* ribozyme [12].

As with base pairs, the key interactions that stabilize base triples are hydrogen bonds. Hydrogen bonds are highly directional and capable of defining specific interactions. Many previous reports discussing base triples consider a wide variety of possible interactions including single hydrogen bond interactions and interactions to non-base components of the nucleotides [11,13]. However, it is clear that multiply hydrogen bonded triples, which consist of at least two hydrogen bonding interactions per base, will be especially stable and the structural conservation of such triples is therefore likely to be significant. They are thus expected to be influential constituents of RNA structure and are therefore of primary interest in this work. Nevertheless, RNA structures are highly dynamic and even triple interactions may be lost as a result of conformational changes. As the number of complex RNA structures increases, the ability to computationally detect and track such changes presents an interesting challenge. A library of 840 theoretically computed base triples has been compiled and is accessible as part of the NAIL (Nucleic Acid Interaction Library) database [14]. Another resource, the NCIR database [13], consists of known non-canonical base interactions including base triples and is compiled through a literature search.

Here, we present a survey, that cross-references the patterns in the NAIL database [14], the Protein Data Bank (PDB) [15] and the records in NCIR [13], and is able to deliver a catalogue of base triples of a specific type in existing crystal structures. We use a graph theoretical approach that is capable of documenting occurrences of NAIL patterns in the PDB, and in doing so, find interactions that are predicted by NAIL but not recorded in NCIR. NCIR is a manual literature search and therefore, such a search is limited to what is reported in the available literature in addition to possibly incomplete coverage due to the manual and labor intensive nature of the compilation process. By employing high tolerances designed to give a ‘fuzzy’ search, our method was also able to retrieve previously unrecorded occurrences of triples contained within the PDB. Surprisingly, we also show that there are multiply hydrogen bonded base triples that occur in the PDB, but were not included in the NAIL dataset. Our investigation of these triples in the large ribosomal subunit structures also revealed conserved interactions that may be essential base interactions in the stabilization of rRNA structure.

## Methods

### RNA search database and query pattern library

The search database was compiled from PDB-sourced RNA and RNA-associated structures, solved by X-ray crystallography to a minimum resolution of 3Å. High resolution structures which became available at a later date, were also included as they became available. Several separate searches using structures solved at lower than 3Å resolution, such as the *E. coli* ribosome at 3.5Å [16], were also done. A library of 942 pattern matrices was generated as queries for the search engine, consisting of (i) The 840 base triple patterns from the Nucleic Acids Interaction Library [14] and (ii) 102 patterns generated by an alternative approach (Additional File 1 Figure S1).

### Searching program and approach

**Figure 1:**
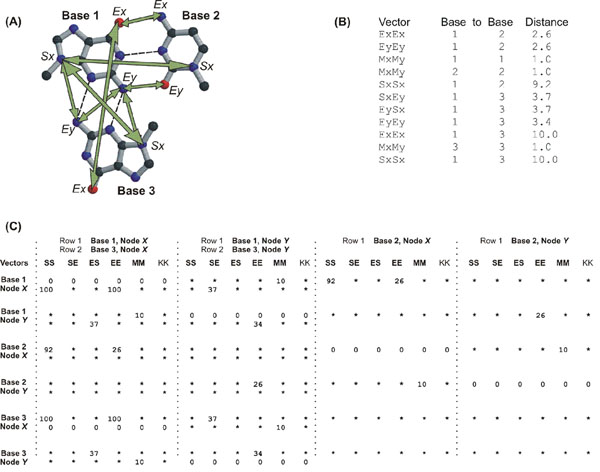
The NASSAM pseudoatom vectors and pattern matrix system. (A) Example of a base triple composed of a guanine (Base 1), a cytosine (Base 2) and another guanine (Base 3). The pseudoatom nodes used to set the distances for the pattern matrix have been marked with *Sx*, *Ex* and *Ey* while the distances between these nodes have been marked with arrows. (B) An example set of vectors and their corresponding distances (in Angstroms) which define the GGC triple orientation in section (A). (C) The corresponding pattern matrix file built from the vectors (distances X10) defined in section (B) for the triple pattern shown in section (A). As an example, the *SxSx* distance between Base 1 and Base 3 is 10Ǻ and is marked 100 under the SS column where Base 1, Node X and Base 3, Node X intersect.

The computer program NASSAM (Nucleic Acid Search for Substructures and Motifs)[17], which uses a simplified vectorial representation of the nucleic acid bases, was used as the search engine and primary screening step. NASSAM implements the Ullmann subgraph isomorphism algorithm [18] for comparing pseudo-atom representations of RNA base orientations. Each of the four RNA bases are represented by two pseudoatom vectors consisting of four pseudoatoms; where one pseudoatom is the start node and another pseudoatom serves as the end node (Figure 1). The search input base triple patterns were created from pseudo-atom distances that hypothetically represent a triple, or they can be created by measuring the distances of the pseudoatoms from an actual occurring formation. Additional pseudo-distances were incorporated into the matrices to ensure that the midpoints of the pseudo-atom vectors for a base were close enough to each other that they were on the same base. These midpoint to midpoint distances internal to a base were constrained to 1Ǻ. NASSAM searches are also not dependent on sequence order. A distance tolerance parameter value, which sets the amount of deviation from the distances supplied in the pattern matrices is also incorporated into a search. This parameter can be supplied as either a discrete distance value, for example 1.7Ǻ, or as a percentage value. Very high distance tolerances (60% and above), which resulted in ‘fuzzy’ searches, were used to ensure that all possible matches were recalled and to facilitate discovery through variations of the original queries. Hits not matching our criteria of two hydrogen bonds per base were eliminated by computing the hydrogen bonding between the three bases in each hit, using the program HBPLUS [19] at default parameters. HBPLUS outputs were screened for matches to our criteria and filtered results were visualized and cross referenced with NAIL, NCIR and available literature.

### Sequence alignments and structure superpositions

Sequence alignments were done using CLUSTALW[20] for 19 prokaryotic 23S rRNA sequences retrieved from selected completed genomes accessible from the NCBI website and four prokaryotic 23S rRNA sequences extracted from PDB structures (Table 1). The same 19 species were used for an alignment of 16S rRNA sequences against two prokaryotic 16S rRNA sequences from structures available in the PDB (Table 1). The chosen species were selected to provide a simple yet diverse representative model of available prokaryotic rRNA sequences. The sequences used in the alignments and their database references are available in Table 1. Structural comparisons of *T. thermophilus* [PDB: 2j01], *E. coli* [PDB: 2awb] and *D. radiodurans* [PDB: 1nkw] to the *H. marismortui* [PDB: 1ffk] structure were carried out using least squares superposition on the phosphate backbone atoms by the program LSQKAB [21] from the CCP4 suite [22]. Geometric descriptions of the triples utilized the nomenclature proposed by Leontis and Westhof [23]. Redundant geometries were identified by ordering the resulting geometries in alphabetical order such as arranging all cis interactions before trans interactions, as well as arranging the interacting edges for each pair alphabetically. Triples with the same geometrical description were then cross checked for differences. The C-H edges of pyrimidines have also been labelled as Hoogsteen edges for uniformity.

**Table 1:** Source of sequences and structures used for sequence-structure comparisons.

Species	Taxonomy: (Super)Phylum	Database code
*Bacteroides fragilis NCTC 9343*	Bacteroidetes/Chlorobi group;	GenBank: NC_003228
*Borrelia burgdorferi B31*	Spirochaetes	GenBank: NC_001318
*Candidatus Protochlamydia amoebophila UWE25*	Chlamydiae/Verrucomicrobia group	GenBank: NC_005861
*Rhodopirellula baltica SH 1*	Planctomycetes	GenBank: NC_005027
*Thiomicrospira denitrificans ATCC 33889*	Proteobacteria	GenBank: NC_007575
*Acidobacteria bacterium Ellin345*	Fibrobacteres/Acidobacteria group	GenBank: NC_008009
*Escherichia coli*	Proteobacteria	PDB: 2awb
*Baumannia cicadellinicola str. Hc*	Proteobacteria	GenBank: NC_007984
*Azoarcus sp. EbN1*	Proteobacteria; Betaproteobacteria	GenBank: NC_006513
*Rhodobacter sphaeroides 2.4.1*	Proteobacteria; Alphaproteobacteria	GenBank: NC_007493
*Anaeromyxobacter dehalogenans 2CP-C*	Proteobacteria; delta/epsilon subdivisions	GenBank: NC_007760
*Anabaena variabilis ATCC 29413*	Cyanobacteria	GenBank: NC_007413
*Dehalococcoides sp. CBDB1*	Chloroflexi; Dehalococcoidetes	GenBank: NC_007356
*Bacillus subtilis subsp. subtilis str. 168*	Firmicutes; Bacilli	GenBank: NC_000964
*Deinococcus radiodurans*	Deinococcus-Thermus; Deinococci	PDB: 1nkw
*Bifidobacterium longum NCC2705*	Actinobacteria	GenBank: NC_004307
*Thermus thermophilus*	Deinococcus-Thermus; Deinococci	PDB: 2j00 / 2j01
*Thermotoga maritima MSB8*	Thermotogae	GenBank: NC_000853
*Aquifex aeolicus VF5*	Aquificae	GenBank: NC_000918
*Sulfolobus solfataricus P2*	Crenarchaeota; Thermoprotei	GenBank: NC_002754
*Pyrobaculum aerophilum str. IM2*	Crenarchaeota; Thermoprotei	GenBank: NC_003364
*Nanoarchaeum equitans Kin4-M*	Nanoarchaeota	GenBank: NC_005213
*Haloarcula marismortui*	Euryarchaeota; Halobacteria	PDB: 1ffk

## Results

### Searching for structural patterns with graph theory

NASSAM [17], a graph theoretical search engine, was used to screen a nucleic acid database for matches to the 840 patterns derived from the NAIL database and a further 102 from our own procedure (see Additional File 1 Figure S1). The results from this primary screen were then filtered for specified hydrogen bonding interactions. The output from this secondary screen, which represent actual occurrences in the PDB, was used to compare and contrast the content of base triple interactions in the NAIL and NCIR databases. The probable importance of particular base triples as conserved motifs was able to be contextually presented by collectively comparing the structural conservation of the hits from our searches and their sequence conservation in a diverse set of sequences. Comparisons of the annotated triples for each structure were integrated to structure superpositions and sequence alignments information to investigate the conservation of triple hits in the prokaryotic ribosomal subunits (Additional File 1 Table S2).

### Comparative overview of triples from the NASSAM search

**Figure 2:**
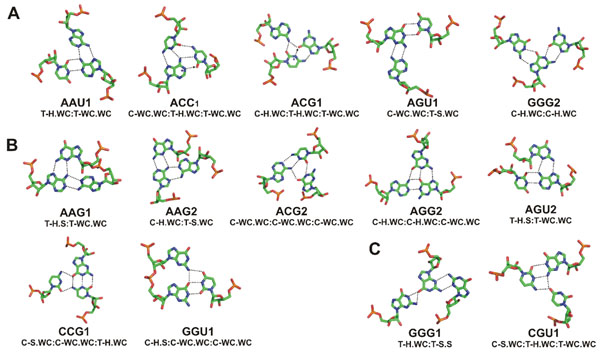
(A) Base triple interactions that were not previously recorded in the NCIR database. (B) Base triple interactions which were not listed in the NAIL library of query patterns but which were found to be present in the NCIR database. (C) Novel triple interactions that were neither recorded in the NCIR database nor listed in the NAIL query dataset. Hydrogen bonds from possibly protonated bases are marked with arrows and + at the protonated donor position. The geometric orientation labels have been abbreviated as: C=Cis glycosidic bond orientation, H=Hoogsteen Edge, T=Trans glycosidic bond orientation, S=Sugar edge, WC=Watson-Crick edge.

This survey using NASSAM found matches for 59 of the total 942 search patterns. Fourteen or approximately 24% of these patterns were discovered for multiply hydrogen bonded base triples that were either not currently represented in NCIR (Figure 2A), not represented in NAIL (Figure 2B) or not found in either resource (Figure 2C). Full details are given in Additional File 1 Table S1. The backbone angles for the residues involved in the novel triples, and the residues before and after them in the sequences, were examined using the Amigos program [24]. No unusual conformations were observed for them. The relative bulk of triples hits retrieved were for occurrences in rRNA structures. This is not unexpected as these are the largest RNA 3D structures available and offer the most opportunity for a diverse array of interactions, including very long range inter-domain interactions. Of the interactions listed in Figure 2, four are currently only found in non-ribosomal structures (ACG1, AGG2, CGU1, GGG2). The majority of the discussion will therefore revolve around triples found in the prokaryotic ribosomal subunits.

### Triple types found in high resolution RNA Structures that are unrecorded in NCIR

On examination of the new triple types in the 23S rRNA of *H. marismortui* and *T. thermophilus* structures, we find that many triples had already been annotated on the secondary structure diagram [1,2]. However, one of the hits AGU1 (Figure 2A) appears to be completely unrecorded in this structure or any other, although it had been theoretically predicted by NAIL. This new triple is a GU (G924.U919) wobble pair in helix 37 in which the guanine is also participating in an AG N3-amino, amino-N1 interaction to A166 [PDB: 1ffk_0] which is situated in a hairpin loop in helix 11. Thus this triple links bases in domains I and II. Domain II accounts for much of the “back” of the ribosomal particle and thus this base triple is far from the functional sites of 23S rRNA. The A166 and G924 positions are conserved in alignment of 23 prokaryotic species sequences. However, in the sequence of *Nanoarchaeum equitans Kin4-M*, an archaeal species, the U919 position is replaced with a cytosine. The ACG1 triple (Figure 2A) was found in only in the two structures of the hepatitis delta virus (HDV) ribozyme [25,26] using a NAIL input pattern which involves a protonated cytosine. Its position between sites for catalysis and protein interaction, may suggest that it contributes to stabilization of the helix backbone necessary for the correct folding of either or both these sites. Another protonated NAIL pattern that finds a match in the database is the ACC1 triple (Figure 2A). This triple, which involves an adenosine protonated at N1, is found in two different locations in the 23S rRNA structures of *H. marismortui*. One of these, the A2485.C2104.C2536 triple, was found to be conserved in our alignments of 23S rRNA sequences. It is within the peptidyl transferase region and is discussed below. The GGG2 triple (Figure 2A), though not listed in NCIR, has been previously identified in a synthetic RNA-DNA hybrid molecule [27], but we find no other examples in naturally occurring structures. NCIR is the result of a manual literature search and therefore, by implication, such a search is limited to what is reported in the available literature in addition to possibly incomplete coverage due to the manual and labor intensive nature of such a compilation process. Furthermore, to our knowledge, NCIR is also not automatically updated as newer structures become available.

### Previously recorded triple types not predicted in NAIL

Seven triple types were found to be recorded in NCIR, but were not predicted in NAIL. This may be because of limitations already discussed by Walberer *et al*.[14], namely: the fixed length of the initial hydrogen bond and problems associated with modeling of non-planar geometries. Thus in the AAG1, AAG2 and AGU2 triples (Figure 2B) a completely planar triple geometry could result in close approaches and possible atomic clashes. The ACG2 triple is found in the *H. marismortui* 23S ribosomal RNA structure and involves a rather non planar association of G2033 to the A1742.C2037 pair. This triple caps a short (4bp) double helix which sweeps up from the end of the double helix to complete the triple. The AGU2 triple (Figure 2B), found in the structure of the *T. thermophilus* 16S rRNA [PDB: 1fjg_A, 1n32_A], consists of an AU reverse Watson-Crick pair and an AG N7 amino, amino N3 pair in a situation where different edges of the shared adenine participates in different non-canonical pairings to form the triple. This triple (A55.G357.U368), is in fact part of a tetrad, listed in NCIR, as G357 also forms a Watson-Crick base pair with C54. In addition to the inter-base hydrogen bonds there is also a hydrogen bond with good geometry between the ribose O2* of G357 and the O2 acceptor of U368. This close approach may be the reason for its exclusion from the NAIL predictions. Another type of triple not predicted in NAIL is CCG1 which occurs in the 5S subunit (Figure 2B) and consists of a Watson-Crick G66.C15 base pair with a bridging C113 [PDB: 1ffk_9]. This interaction forms a junction between three double helices. The GGU1 triple was found to be present in aspartate-tRNA structures as well as 23S rRNA structures (Figure 2B). In the tRNA structures, this triple caps the anticodon stem, stacking with the A24.U11 base pair which in turn stacks on a group of three triples (U12.A23.A9;Ψ13.G22.A46; A14.A21.U8), the triple being augmented by a hydrogen bond between the O2* of residue 45 and the O3* of residue 9. The other occurrence of GGU1 is in the 23S ribosomal RNA structures [G2092.G2093.U2652, PDB: 1ffk_0] where the two guanosines are sequential and form a platform-like structure. This triple caps a stack of two other triples: G2094.A2649.C2651 and A2095.A2612.U2650.

### Completely novel base triple interactions

A further two triple types, CGU1 and GGG1 (Figure 2C) were neither predicted in the NAIL nor listed in the NCIR databases. The CGU1 triple consists of a reverse Watson-Crick GC base pair G515.C548 with a bridging O4 from U519. The triple caps a succession of base triples formed by residues 512-515 and 521 to 523 in *T. thermophilus* tRNA-Gln in complex with its cognate tRNA synthetase [28] [PDB: 1g59_B: C512.C509.G523; U513.A546.G542; A514.U508.A521]. The second novel triple type, GGG1 (Figure 2C), was found in two structures of *H. marismortui* 23S ribosomal subunits [PDB: 1k9m, 1kd1 [29], 1m90 [30]] but not in the other *H. marismortui* high resolution rRNA structures searched. The GGG1 triple type consists of a G.G N3-amino symmetric base pair between G512 and G487. The O6 atom of G512 accepts two hydrogen bonds from N1 and N2 of G504 to complete the triple. This triple can be further extended into a quadruple interaction, which is unlisted in NCIR, via a Watson-Crick pairing between G487 and C515. In some other 23S structures, G512 and G487 are further apart and do not hydrogen bond, although in all the structures, hydrogen bonding occurs from G512 N2 to the O4* in the ribose of G487. The interaction is sandwiched between another triple U488.G503.A513 and a tetrad A485.A509.C505.U481.

## Discussion

### Base triples as constituents of interactions between RNA secondary structures

Many of the base triples found contain a Watson-Crick pair. Thus, approximately 55% of the triples found in *H. marismortui* 23S rRNA, contain a Watson-Crick pair. Triples were observed as interactions involved in RNA helix packing, or at interfaces of RNA secondary or tertiary structure interactions. A listing of interactions between RNA secondary structure elements highlights the frequency of interactions involving at least one loop structure of any type interacting with either another loop or a helix (Additional File 1 Table S2). These interactions outnumber helix-helix and intra helix interactions. We further observed that the same triple types occur in different, larger structural motifs involving a variety of RNA secondary structures. For example, there are six AGC amino-N3, N1-amino; Watson-Crick triples in *H. marismortui* 23S rRNA, which are variously involved in helix to internal loop interactions, hairpin loop – hairpin loop – hairpin loop interactions, helix to hairpin loop interactions and helix to multi-branched loop interactions (Additional File 1 Table S2). Furthermore, three of these six occurrences are also involved in inter-domain interactions. One of these six AGC triples interfaces domain IV and domain VI and is conserved in the sequence alignments, while the other five show varying degrees of conservation.

Two occurrences of the common AGC amino-N3, N1-amino; Watson-Crick triples were also observed, in *T. thermophilus* 16S rRNA, one of which is also involved in an inter-domain interaction. Of the two AGC amino-N3, N1-amino; Watson-Crick triples, one involves bases in helices H8 and H14 (G347.C342.A160) and the other bases in helices H12 and H21. The helices for the first triple end in a GAAA (GNRA) tetraloop and a UACG (UNCG) tetraloop, respectively. The interaction between these loops has been previously noted to be an unusual packing arrangement[2]. The second triple is between the second A (A608) of the GAAA loop and the C.G (C308.G292) base pair, which closes the UACG loop. Interestingly, this second example in 16S RNA, the adenine base, although not part of a GNRA tetraloop, is in a GAAAG internal loop sequence where the GAAA has a very GNRA like conformation. The significance of this may purely be that this base in the GNRA conformation is accessible and has both faces available for tertiary binding. This position may be particularly important for the interaction of tetraloop structures with other features.

### The prokaryotic ribosomal subunits: occurrences and conservation of base triples

**Figure 3:**
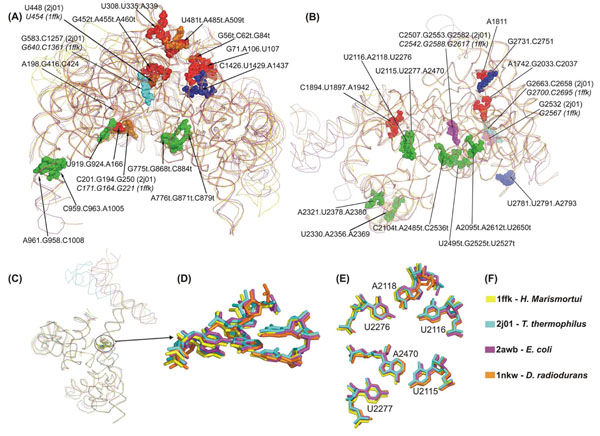
Base triples conserved in four 23S rRNA structures. (A) Triples from domains I (red), II (green) and III (blue) highlighted in spacefilling mode and numbered using the *H. marismortui* numbering unless stated otherwise. (B) Triples in domain IV (red), domain V (green) and domain VI (blue). Triples not originally detected in the *H. marismortui* structure which were detected in the *T. thermophilus* search and were found to have conserved equivalent interactions in the other three structures are also highlighted in section (A) as orange spacefills for domain I and cyan spacefills for domain II, and in section (B) as magenta spacefills for domain V and cyan spacefills for domain VI. (C, D) A stacked triples motif located on a junction in domain V which joins three subdomains in four of the available structures compared. (E) shows the two stacked triples, separated for clarity, consist of two planar UAU Hoogsteen, Watson Crick triples. The colors used for each of the 23S subunits compared are presented in section (F).

Many of the interactions in the *H. marismortui* 23S and *T. thermophilus* 16S ribosomal RNA discussed here, have been previously noted in the structure-based secondary structure diagrams available [1-3]. Not all the triples discussed here are novel observations, but our approach in inventorizing specific classes of triple puts the collective contribution of these structural formations into perspective (Additional File 1 Table S2). We have found 28 triples in *H. marismortui* 23S rRNA, and the 23S rRNA sequence alignments for 23 prokaryotic species in Table 1, showed that the base components for twelve of these triples are totally conserved across the aligned sequences (Additional File 1 Table S2). Domain V, which contains the peptidyl transferase center has the largest number of triples fitting our criteria, followed by domain II. A comparison of triples in four 23S rRNA structures available (*H. marismortui*, *D. radiodurans*, *E. coli*, *T. thermophilus*; PDB: 1ffk, 1nkw, 2awb, 2j01 respectively) and using the *H. marismortui* structure as a reference, shows that 21 out of the 28 triples are also conserved in the other three structures. We were further able to observe that there appear to be stackings and clusterings of these triples (Figure 3). A similar clustering of the A-minor motif classes of nucleotide triples into ‘A patches’ has been previously reported [31]. Our observations strengthen the indication that highly hydrogen bonded and stable interactions are clustered together thereby implying a requirement for structural rigidity or integrity in the regions where they are most observed. For example, a novel observation of two stacked UAU Hoogsteen, Watson-Crick triple motifs are discussed below. Another stacked pair of base triples in the large ribosomal subunit (C959.C963.A1005 and A961.G958.C1008) may possibly be involved in maintaining the structure of an internal loop which disrupts the middle of the long helix 38 and is conserved in all our 23 aligned sequences. However, in contrast to the high conservation of triples in the large subunit, our alignments for the 16S rRNA sequences, which are otherwise well known to be highly conserved in sequence [32], showed that only three out of the fifteen triples in *T. thermophilus* [PDB: 1fjg] are conserved for all three base positions of a triple. Taking into account occurrences for both subunits, these observations suggests that triples may also be possibly used as an opportunistic interaction mechanism for increasing sequence diversity while preserving the general fold of the ribosomal RNA assemblies and is further discussed in the following sections.

### Geometric families for 23S rRNA base triples

The conservation of geometric orientation for a triple position to an extent enables the conservation of 3-D space for a triple and thus preserving the backbone conformation of an RNA molecule despite variations in the nucleotide sequence. An analysis of the interaction geometry for 23S rRNA triples was used to investigate occurrences of repeats for base geometry and the resulting effect such conservation may have on sequence variation while still preserving the general conformation of the sugar-phosphate backbone. 23S rRNA triples from *H. marismortui* and *T. thermophilus* 23S rRNA structures were described using the nomenclature proposed by Leontis and Westhof [23]. Triples of equivalent positions in the *E. coli* and *D. radiodurans* structures identified via the structural alignment were also described using the same nomenclature. The sequence alignment data was then integrated to this information and the variability of base sequence or content for these triples could be observed.

All the triples observed in this survey contain at least one base which uses its Watson-Crick edge to interact with other bases. The most common triple in 23S rRNA is the AGC triple from the Cis S / WC – Cis WC / WC geometric family (Additional File 1 Table S3). In the 23S rRNA structure, this geometry appears to be exclusive to the AGC triple. However, the most common geometric family in the 23S subunit is the Cis WC / WC – Trans H / WC family which has seven examples. These seven examples occur in triples with three different base compositions, ACU, AUU and CGG (Additional File 1 Table S3). The majority of triples occur as composite base pairs where one base interacts via two of its edges to the other two bases of the triple. However, four cases were observed where two bases are interacting with two edges (ACG, CGG, GGU, GUU - Additional File 1 Table S3). All four of these cases are unique and are not repeated in the 23S rRNA structure.

### 3D space conservation in triples with unconserved base content

**Figure 4:**
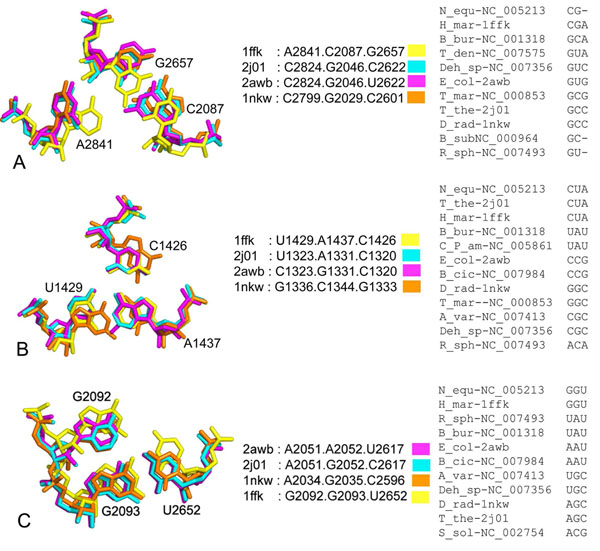
Least squares superpositions of *H. marismortui* triples that were not conserved in sequence with the three other structures (*D. radiodurans*, *E. coli*, *T. thermophilus*,). These show maintenance of equivalent structural spaces where (A) documents possibly organism specific domain interfacing interactions; (B) interactions where variation occurs as a whole triple; (C) interactions where most variations may have gone through a single base mutation step. The equivalent triples selected from the alignment of 23 different prokaryotic species from Table 1 are shown to the right of each corresponding triple superposition.

Structure comparisons between the four 23S rRNA structures used showed that several triple locations have variable base content, although the equivalent base positions superpose well and occupy a similar structural space (Figure 4). In some cases, this observation can be attributed to unique interactions, such as inter-domain interactions, which may vary between different species while still maintaining the general fold of the phosphate backbone containing these bases (Figure 4A). For all three cases presented in Figure 4, the 3D space occupied is approximately similar for each case and does not appear to shift the phosphate backbone drastically despite the variety of base combinations. The geometric orientation for structurally equivalent triples with conserved 3D space, are conserved in almost all the cases (Figure 4). In some cases, the geometric orientation of an interaction is not conserved due to the equivalent bases not interacting via hydrogen bonds as per the criteria of this survey such as in Figure 4A or may have an additional interacting edge such as the case for U2652 of the GGU triple in Additional File 1 Table S3 and Figure 4C.

Sequence variation has also been observed at points of inter-domain interactions. These triples are quite possibly the result of opportunistic interactions resulting from the placement of the component nucleotides by the conservation of the sugar-phosphate backbone fold. These opportunistic interactions are therefore expected to vary between different species and therefore the geometric family of the interactions is also not expected to be conserved. One such example is the A2841.C2087.G2657 triple in *H. marismortui* (Figure 4A) which has equivalently placed bases in the *E. coli*, *T. thermophilus* and *D. radiodurans* structures that however differ in sequence. This is an interaction between domains V and VI where the geometry of interaction for positions equivalent to A2841 (DVI) are not conserved. As previously noted, the formation of triples that are unconserved in sequence can be seen as an opportunistic mechanism for effecting sequence diversity while still preserving the backbone conformation of the structure in general.

### A stacked UAU Hoogsteen, Watson-Crick triple motif in prokaryotic 23S rRNA

One interesting highly conserved occurrence in prokaryotic 23S rRNA is the presence of two UAU Hoogsteen, Watson Crick interactions stacked on each other. These triples [U2116.A2118.U2276 and U2115.A2470.U2277; PDB: 1ffk_0] have been previously recorded in NCIR. However, when viewed together the adenines appear stacked in opposite positions to each other (Figure 3C). This same stacked motif was also found in our survey of the *E. coli* [PDB: 2awb], *T. thermophilus* [PDB: 2j01] and *D. radiodurans* [PDB: 1nkw] 23S rRNA structures. The superposition for the formations in all four structures shows good structural conservation. To our knowledge, there have been no previous discussions or hypotheses with regard to the possible functions of this novel stacked UAU Hoogsteen, Watson Crick triples motif. This motif is situated at a junction which joins the three subdomains of domain V (Figure 3C). One of these three subdomains form the binding site for protein L1, another forms the majority of the central protuberance region and the third extends to the direction of domain VI and the putative peptidyl transferase active site [1]. It has been previously observed that the U2115.A2470.U2277 triad is stacked below a UGCAG pentad[33] (U2278.G2471.C2114.A2633.G2630). Our results demonstrate that there are actually two structurally conserved UAU triples situated at a multi-loop junction stacked to the UGCAG pentad. At present, the only other occurrence of this kind of UAU Hoogsteen, Watson Crick triple in a non-23S rRNA structure is a lone triple in the structure of cysteinyl tRNA synthetase[34] [PDB: 1u0b].

### Interactions between rRNA domains

More than 80% of the triples in both the large ribosomal subunit and in the 16S rRNA mediate intra-domain interactions. However, inter-domain interactions involving bases very distant to each other in the polynucleotide sequence were also observed. Two interactions that interface three of the six domains and three which interface two domains were observed in the large subunit structure (Figure 3A, 2B). In the first three-domain triple, a G.C Watson-Crick and A.G N3-amino, amino-N1 triple (G2449.C418.A1921) forms an interaction between domains I, IV and V (Additional File 1 Table S2). Domain IV constitutes much of the subunit interface in contact with the 30S particle and helices 67 to 71 form an area around the putative active site cleft on the subunit interface side of the 23S rRNA[1]. The adenine in this triple is in helix 68 and appears to be located in a relatively variable region in our alignment. This is the only example we have observed which shows the interactions of three bases, each on a separate hairpin loop and on different domains. The second triple that interfaces three different domains is U1371.A2054.U2648. U1371 on a multi-branched loop in domain III hydrogen bonds with the A2054 in a multi-branched loop on domain IV, which in turn is hydrogen bonded to U2648 on a small internal loop in domain V [1]. An interesting difference in the structural organization of the two ribosomal subunits is that while the domains of the 16S rRNA each form distinct components, the domains of 23S rRNA are much more intricately linked together, which has been suggested may reflect the lesser requirement for flexibility in this subunit[1]. This is also reflected in our work, where multiply hydrogen bonded triples which are components of inter-domain interactions are more numerous in the 23S rRNA than the 16S rRNA subunit. We did not observe any triples that involved all three domains of the *T. thermophilus* 16S rRNA structure [PDB: 1fjg], although two triples were observed to interface two different domains. One example, A608.C308.G292, has been previously discussed. The other appears to be a U13.U20 in a hairpin loop interacting with A915, where the Watson-Crick face of U20 forms hydrogen bonds with both U13 and A915.

### Base triples and links to ribozymic activity

Domain V of the *H. marismortui* 23S rRNA has been investigated for links to its ribozymic peptidyl transferase activity [35]. There are 11 interactions that involve base triples in this domain. One cluster of interactions involves the double UAU Hoogsteen, Watson Crick discussed previously. Another set of base triples forms interactions on stems leading or exiting the multi-branched loop (helices 73, 89, 90) where the A-loop [36] is located. These include a triple [C2104.A2485.C2536; PDB: 1ffk] [1] that was also found to be structurally conserved in the structural alignments and is also conserved in all the sequences aligned. This ACC triple is adjacent to A2486, which was originally hypothesized as the peptidyl transferase acid-base catalytic residue by Nissen *et al*.[35], although subsequent work has shown that peptide bond formation in the peptidyl transferase centre does not involve this acid-base catalysis mechanism[37-40]. The A2450.C2501 component of this triple (*E. coli* numbering used), is also involved in an A-minor interaction with A76 of P-site tRNA[41].

Triples in the vicinity of other ribozyme active sites have previously been observed; an example being the base triple sandwich in the structure of the *Tetrahymena* ribozyme[12], where the 3’-terminal guanosine (ωG), which serves as the attacking group for RNA cleavage, participates in a triple interaction with the G264.C311 base pair. This triple fits the multiply hydrogen bonded criteria of this survey and is in turn sandwiched by three other base triples. Of the 3 triples in the sandwich, the A263.C262.G312 interaction above the ωG triple, is also of the type covered in this survey. Both triples were found by a NASSAM search although they were not in the original search due to the resolution of the *Tetrahymena* ribozyme structure [3.8Å, PDB: 1x8w] being lower than our cut-off point. The inter-domain interactions and helix stabilization functions carried out by base triples may be important, albeit collective factors, in determining the correct folding of the RNA molecule to enable its catalytic functions.

### The ribosomal polypeptide exit tunnel

**Figure 5:**
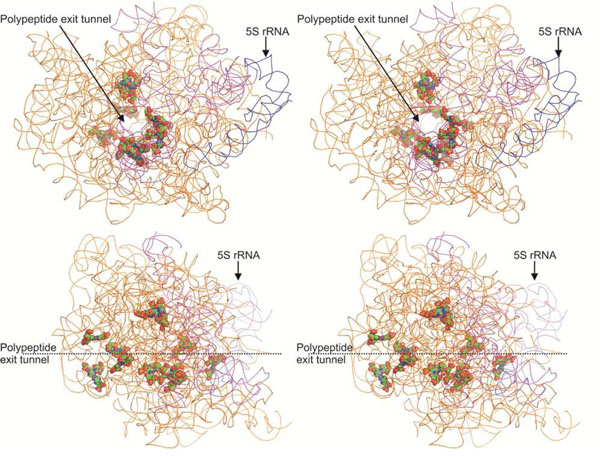
Stereo diagrams showing the eleven triples that approach the polypeptide exit tunnel in *H. marismortui* 23S rRNA (PDB: 1ffk). The triples are shown in space filling mode viewed looking down the polypeptide exit tunnel (above) and in an orthogonal view (below). To aid orientation, 5S rRNA has been colored in blue while domain V of 23S rRNA has been colored magenta.

The polypeptide exit tunnel is the passage through which nascent proteins pass as they are synthesized. This tunnel begins immediately below the peptidyl transferase center, and is approximately 100Å in length[42]. We found that a total of 32 bases, which are the components of 11 triples, are in positions that were defined by Nissen *et al.*[35] as approaching the tunnel (Figure 5). This includes U1371.A2054.U2648 [PDBID 1ffk], which, as discussed earlier, links domains III, IV and V, although the other ten triples do not participate in any inter-domain interactions. Almost one-third of the triples in the large ribosomal subunit structure of *H. marismortui* take part in interactions close to the tunnel, including the ACC triple adjacent to the hypothesized peptidyl transferase center discussed in the previous section. Multiply hydrogen bonded base interactions may contribute significantly towards the conformational integrity of the tunnel.

### Possible exclusivity of interactions to particular structures

Several types of triples appear to occur more than once within one structure, but are not presently seen in any other class of RNA structure. For example, the new ACC1 triple (Figure 2A) occurs at two different positions in the large ribosomal subunit but does not occur in any other RNA structure used in our search. The two CCG triples found, C37.G43.C46 and C15.G66.C113 [PDB: 1ffk_9], were found only in the 5S rRNA of the large ribosomal subunit. Several of the interactions found have only been observed at the same position in a particular structure, and do not occur anywhere else in that structure or any other structure. Moreover, the ribosomal structures are extremely large compared to the other RNA structures and are likely to contain a wider diversity of triples, and therefore these observations, although inconclusive, are noted for the record.

### Future extensions to the NASSAM program - roles of RNA backbone components, water molecules and metal ions

At present the methodology is restricted to base-base interactions. Although this has revealed new types of interactions, it is clear that base to backbone interactions are also of great importance[43]. While inclusion of backbone information will pose some challenges for the graph theoretical methodology as it greatly increases the number of nodes that can potentially be matched, this is nevertheless an important future enhancement that can be added. Another important extension that can be envisaged is the addition of information relating to water molecules and metal ions (especially Mg^2+^ ions) which play important roles in the stabilization of interbase interactions[44]. Here there is another problem in that it is extremely difficult to accurately define waters and low atomic number metal ions (such as Mg^2+^) in crystal structures unless they are at least 2.0Å in resolution; very few RNA structures match this criterion. This problem has been discussed, for example, by Banatao et al (2003) [44], who observed that many metal sites are mislabelled or completely missing in RNA structures. Nevertheless, with ribosomal structures (such as PDB: 1vqs) already at 2.2 Å, this kind of analysis may soon be an accessible goal, and would clearly greatly increase the number of possible interaction patterns beyond those discussed here.

## Conclusions

The results of our survey reveal that multiply hydrogen bonded base triples have a high degree of conservation between comparable ribosomal structures, therefore suggesting significant collective contributions towards the overall folding and stabilization of the RNA molecule. By annotating triple patterns and correlating theoretically predicted base triples from NAIL with the literature-based compilations in the NCIR database, we were able to discover a number of multiply hydrogen bonded base triple formations that had not been previously recorded and/or had not been predicted theoretically. The same annotation approach has enabled motif discovery by putting into context known patterns of interactions such as the stacked UAU Hoogsteen, Watson Crick motif. The value of expertly curated databases such as NCIR [13] and SCOR [45] are indisputable. Computationally generated libraries such as NAIL[14] have proven to be an important resource towards the discovery of new base interactions. Computational structure annotation methods, which can quickly locate tertiary interactions of a given type, large or small, can be invaluable for the comparison of complex structures as well as for increasing the coverage and volume for manual curation. This capability provides the foundation for further experimental work in investigating the specific contributions from possibly essential base interactions which can in turn correlate RNA tertiary structure to their corresponding function. As an outcome of this work, we have made available the NASSAM program via a web enabled interface at http://mfrlab.org/grafss/nassam/.

## Competing interests

The authors declare that they have no competing interests.

## Authors’ contributions

MFR built the search database, designed the research methodology, carried out the base triple searches, analysed the data and drafted the manuscript. AMH designed the alternative triple pattern approach and carried out the initial NASSAM optimizations. PW participated in the design and coordination of the work. PJA conceived the study, wrote the algorithm, participated in the design, coordination and drafting of the manuscript. All authors read and approved the final manuscript.

## Supplementary Material

Additional File 1 Supplementary Figure S1 and Supplementary Tables S1-S3 in PDF format. Click here for file
