# Supplementary material for: Novel base triples in RNA structures revealed by graph theoretical searching methods
Source: BMC Bioinformatics. 2011 Nov 30;12(Suppl 13):S2. doi: 10.1186/1471-2105-12-S13-S2 (PMC3278836; doi:10.1186/1471-2105-12-S13-S2)
Supplement: Additional File 1 — Supplementary Figure S1 and Supplementary Tables S1-S3 in PDF format. [file 1471-2105-12-S13-S2-S1.pdf]

Supplementary Figure and Tables

Figure S1. Methodology for creating a possible base triple formation by superimposing a shared base between two base pairings as described by Tinoco and co-workers(1); section A shows an example where superimposition of the shared base residue does not result in a plausible triple formation while section B is an example where the superimposition of the shared base results in a possible formation which is then used as a NASSAM input pattern.

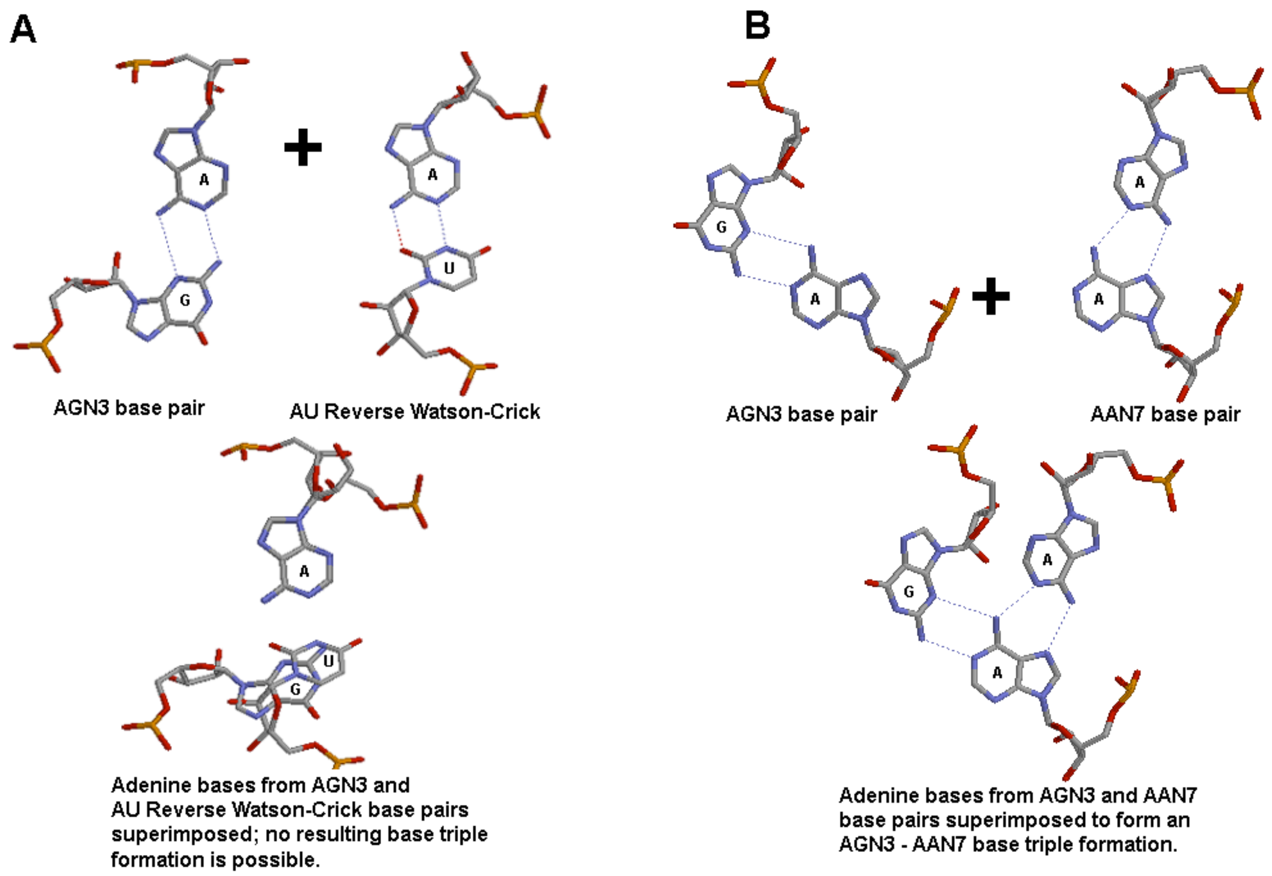

Table S1. Full details of the new triple patterns shown in Figure 1. (A) Base triple interactions which were not previously recorded in the NCIR(2) database. (B) Base triple interactions which were not listed in the NAIL library(3) of query patterns but which were found to be present in the NCIR database. (C) Novel triple interactions which were neither recorded in the NCIR database nor listed in the NAIL query dataset. The primary comparative measure to differentiate triples were the hydrogen bonding interactions involved. Triples in NCIR may at times have different interactions visually presented although they are generally accepted as the same with those calculated by HBPLUS(4) in cases where the interacting residues are the same as noted here.

**A.**	
**AAU1** (NAIL output number = 01567)<br>1n32_A: U827.A860.A872<br>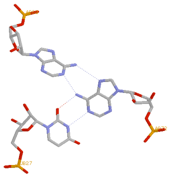	**ACC1** (NAIL output number = 30007)<br>1ffk_0, 1jj2_0, 1k8a_A, 1k9m_A, 1k9m_A, 1m90_A, 1n8r_A, 1nji_A, 1q81_A, 1q82_A, 1q86_A, 1qvg_0, 1s72_0: C2104.A2485.C2536<br>1ffk_0,1jj2_0,1k8a_A,1k9m_A,1k9m_A, 1m90_A, 1nji_A, 1q86_A, 1qvg_0, 1s72_0:C959.C963.A1005<br>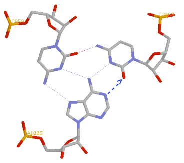<br>Hydrogen bond resulting from possibly protonated adenine marked with blue dotted arrow. NCIR entry disregards hydrogen bonding donated from a possibly protonated A N1.
**ACG1** (NAIL output number = 14213)<br>1drz_B, 1vc7_B: C141.A143.G161<br>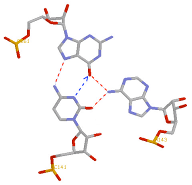<br>Hydrogen bond from possibly protonated cytosine marked with blue dotted arrow.	**AGU1** (NAIL output number = 04298)<br>1ffk_0, 1jj2_0, 1k8a_A, 1k9m_A, 1k9m_A, 1m90_A, 1n8r_A, 1nji_A, 1q81_A, 1q82_A, 1q86_A, 1qvg_0, 1s72_0: A166.U919.G924<br>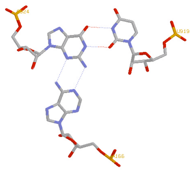
**GGG2** (NAIL output number = 06767)<br>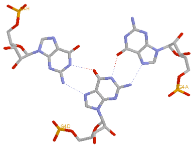<br>Repeat hits in 1n7a, 1n7b (both structures are synthetic RNA-DNA hybrids used for studying radiation induced phasing) Interaction not recorded in NCIR but observed by Ravelli *et al.*(5)	
**B.**	
**AAG1**<br>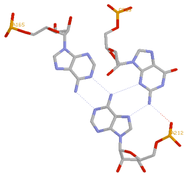<br>1jj2_0, 1k8a_A, 1k9m_A, 1k9m_A, 1q81_A, 1q86_A, 1qvg_0, 1s72_0: A1414.G1681.A1682<br>1jj2_0, 1k8a_A, 1k9m_A, 1k9m_A, 1m90_A, 1n8r_A, 1q81_A, 1q82_A, 1q86_A, 1qvg_0, 1s72_0: .A486.G506.A511<br>1m90_A, 1q81_A: G482.A486.A511<br>1lng_B: A165.G209.A212	**AAG2**<br>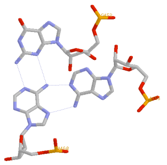<br>1ffk_0, 1jj2_0, 1k9m_A, 1m90_A, 1q81_A, 1s72_0: G452.A455.A460<br>1fjg_A: A978.G1316.A1360<br>1mzp_B: A10.A15.G39<br>1u8d_A: A33.G38.A66
**ACG2**<br>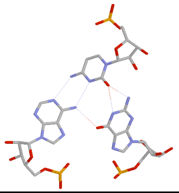<br>1ffk_0, 1jj2_0, 1k8a_A, 1k9m_A, 1k9m_A, 1m90_A, 1n8r_A, 1nji_A, 1q81_A, 1q82_A, 1q86_A, 1qvg_0, 1s72_0: A1742.G2033.C2037	**AGG2**<br>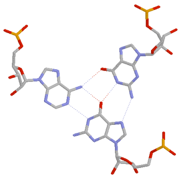<br>1fjg_A, 1n32_A: G64.G68.A101<br>1u9s_A: G177.G188.A192
**AGU2**<br>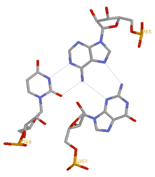<br>1fjg_A, 1n32_A: .A55.G357.U368	**CCG1**<br>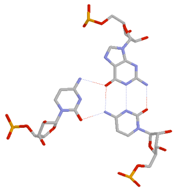<br>1ffk_9, 1jj2_9, 1s72_0: C15.G66.C113<br>1k8a_B, 1k9m_B, 1m90_B, 1n8r_B, 1nji_B, 1q82_B: C3015.G3066.C3113

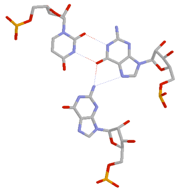 **GGU1**<br>1asy_S/R, 1asz_S/R: G610.U625.G645<br>1efw_C/D, 2tra, 3tra: G10.U25.G45<br>1il2_D: G1910.U1925.G1945<br>1il2_C: G910.U925.G945<br>1ffk_0, 1fg0, 1jj2_0, 1k8a_A, 1k9m_A, 1k9m_A, 1m90_A, 1n8r_A, 1nji_A, 1q81_A, 1q82_A, 1q86_A, 1qvg_0, 1s72_0: G2092.G2093.U2652	
**C.**	
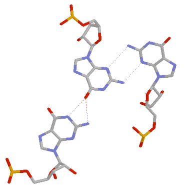 **GGG1**<br>1k9m_A, 1m90_A, 1kd1_A: G487.G504.G512	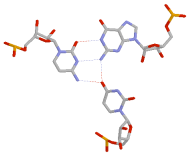 **CGU1**<br>1g59_B/D, 1n77_C/D, 1n78_C/D: G515.U519.C548

Table S2. A listing of the triples conserved in prokaryotic 23S rRNA with the *H. marismortui* structure as the reference comparison (PDBID = 1ffk_0) and triples in the T. thermophilus 16S rRNA (1fjg). The secondary structure descriptions utilized for 23S rRNA were based on the secondary structure diagrams in Ban *et al.*(6).

1ffk_0 Triples in the 23S subunit from *H. marismortui* as primary structure reference.
Domains I-VI are abbreviated as DI to DVI; multi loop = multi-branched loop;
[t] Bases approaching polypeptide exit tunnel previously identified in literature(7).
Base positions which were observed as conserved when aligned with 22 other prokaryotic 23S sequences are underlined in the *H. marismortui* triples.

Triple	NASSAM hit and observed secondary structure interaction	Equivalent positions through structural alignment
**AAG**	DI (G452[t].A455[t].A460[t])<br>*Internal multi loop interactions.*	(G446.A449.A454) 2awb_B / 2j01_A<br>(G458.A461.A466) 1nkw_0
**AAU**	DV (A2095[t].A2612[t].U2650[t])<br>*Helix A.U to A2612 on another helix.*	(A2054.A2577.U2615) 2awb_B / 2j01_A<br>(A2037.A2556.U2594) 1nkw_0
	DV (A2321.U2378.A2380)<br>*Internal multi loop interactions.*	(A2287.U2344.A2346) 2awb_B / 2j01_A<br>(A2266.U2323.A2325) 1nkw_0
	DI (U481[t].A485[t].A509[t])<br>*Interactions within large internal loop.*	(C475.A479.A503) 2awb_B<br>(U475.A479.A503) 2j01_A<br>(U486.A490.A513) 1nkw_0
	DV (U2330.A2356.A2369)<br>*Helix A.U to A2369 in internal loop.*	(U2296.A2322.A2335) 2awb_B / 2j01_A<br>(U2275.A2301.A2314) 1nkw_0
	DI (A479.U499.A503)<br>*NASSAM hit in 2j01_A*	(A485.C505.A509) 1ffk_0<br>(A479.U499.A503) 2awb_B<br>(A490.U509.A513) 1nkw_0
	DIII (A1308.A1608.U1621)<br>*NASSAM hit in 2j01_A*	(A1414.G1683.U1696) 1ffk_0<br>(A1308.A1608.U1621) 2awb_B<br>(A1321.A1624.U1637) 1nkw_0
**ACC**	DV (C2104[t].A2485[t].C2536[t])<br>*Internal multi loop interactions.*	(C2063.A2450.C2501) 2awb_B / 2j01_A<br>(C2046.A2429.C2480) 1nkw_0
	DII (C959.C963.A1005)<br>*Interactions within internal loop.*	(C865.C868.A909) 2awb_B / 2j01_A<br>(C878.C881.A921) 1nkw_0
**ACG**	DIV (A1811). DVI (G2731.C2751)<br>*Helix G.C (DVI) interacting with A in internal loop (DIV).*	(A1755). (G2694.C2715) 2awb_B / 2j01_A<br>(A1746). (G2673.C2695) 1nkw_0
	DIV (A1921). DV (G2449). DI (C418)<br>*Hairpin loop - hairpin loop - hairpin loop interactions.*	No similar triple interactions.
	DI (A198.G416.C424)<br>*Helix G.C to A in hairpin loop.*	(A227.G410.C417) 2awb_B / 2j01_A<br>(A204.G423.C430) 1nkw_0
	DVI (A2841). DV (G2657.C2087)<br>*Helix G.C to A in hairpin loop.*	(C2824). (U2622). (G2046) 2awb_B<br>(C2824). (C2622). (G2046) 2j01_A<br>(C2799). (C2601). (G2029) 1nkw_0
	DII (A776[t].G871[t].C879[t])<br>*Helix G.C (between hairpin and multi loops) to multi loop.*	(A685.G778.C786) 2awb_B / 2j01_A<br>(A698.G791.C799) 1nkw_0
	DII (A961.G958.C1008)<br>*Helix G.C to A in internal loop.*	(A866.G864.C912) 2awb_B / 2j01_A<br>(A879.G877.C924) 1nkw_0
	DIV (A1742.G2033.C2037)<br>*A on bulge, C on bulge (same stem) to G in multi loop.*	(A1664.G1992.C1996) 2awb_B / 2j01_A<br>(A1681.G1975.C1979) 1nkw_0
	DI (A283.C426.G266)<br>*NASSAM hit in 2j01_A*	(-.C433.G237) 1ffk_0<br>(-.C426.G266) 2awb_B<br>(-.C439.G243) 1nkw_0
**ACU**	DIV (C1894.U1897.A1942)<br>*Intra helix interaction (helix A.U to C on bulge loop).*	(C1838.U1841.A1901) 2awb_B / 2j01_A<br>(C1830.U1833.A1884) 1nkw_0
	DII (U917.A926.C1043)<br>*Helix A.U to C in multi loop.*	(U824.A833.C944) 2awb_B<br>(A824.U833.G944) 2j01_A<br>(U837.A846.G955) 1nkw_0

	DIII (C1426^{t}.U1429^{t}.A1437)<br>*Helix A.U to C in internal loop*	(C1320.C1323.G1331) 2awb_B<br>(C1320.U1323.A1331) 2j01_A<br>(G1333.G1336.C1344) 1nkw_0
**AGU**	DI (A166). DII (U919.G924)<br>*Helix G.U to A in hairpin loop.*	(A196). (U826.G831) 2awb_B / 2j01_A<br>(A173). (U839.G844) 1nkw_0
**AUU**	DIII (U1371^{t}). DIV (A2054^{t}). DV (U2648^{t})<br>*A and U in multi loop to U2648 in small internal loop.*	(U1267). (A2013). (U2613) 2awb_B / 2j01_A<br>(U1280). (A1996). (U2592) 1nkw_0
	DI (U308.U335.A339)<br>*A and U in hairpin loop to U308 on helix bulge.*	(U302.U328.A332) 2awb_B / 2j01_A<br>(U313.U339.A343) 1nkw_0
	DV (U2116.A2118.U2276)<br>*Intra-helix interaction.*	(U2075.A2077.U2243) 2awb_B / 2j01_A<br>(U2058.A2060.U2222) 1nkw_0
	DV (U2115.U2277.A2470)<br>*Internal multi-loop interactions.*	(U2074.U2244.A2435) 2awb_B / 2j01_A<br>(U2057.U2223.A2414) 1nkw_0
	DVI (U2781.U2791.A2793)<br>*Helix A.U to U2791 in hairpin loop.*	(U2689.U2756.A2758) 2awb_B / 2j01_A<br>(U2726.U2736.A2738) 1nkw_0
	DII (U562). (A2033.U2028)<br>*NASSAM hit in 2j01_A*	(U619).(A2074.U2069) 1ffk_0<br>(U562).(A2033.U2028) 2awb_B<br>(U571).(A2016.U2011) 1nkw_0
**CGG**	DII (G775^{t}.G868^{t}.C884^{t})<br>*Internal multi loop interactions.*	(G684.G775.C791) 2awb_B / 2j01_A<br>(G697.G788.C804) 1nkw_0
	DII (G702.C726.G744)<br>*Helix G.C to G744 in multi loop / Internal multi loop interactions.*	No similar triple interactions.
	DI (G56^{t}.C62^{t}.G84^{t})<br>*G and G in multi loop to C in hairpin loop.*	(G60.C66.G88) 2awb_B / 2j01_A<br>(G59.C65.G87) 1nkw_0
	DI (C201.G194.G250)<br>*NASSAM hit in 2j01_A*	(C171.G164.G221) 1ffk_0<br>(C201.G194.G250) 2awb_B<br>(C178.G171.G227) 1nkw_0
	DV (C2507.G2553.G2582)<br>*NASSAM hit in 2j01_A*	(C2542.G2588.G2617) 1ffk_0<br>(C2507.G2553.G2582) 2awb_B<br>(C2486.G2532.G2561) 1nkw_0
	DV (C2368.G2349.G2382)<br>*NASSAM hit in 2j01_A*	(C2405.G2383.U2419) 1ffk_0<br>(C2368.G2349.G2382) 2awb_B<br>(C2347.G2328.G2361) 1nkw_0
	DV (G2532). DVI (G2663.C2658)<br>*NASSAM hit in 2j01_A*	(G2567). (G2700.C2695) 1ffk_0<br>(G2532). (G2663.C2658) 2awb_B<br>(G2511). (G2642.C2637) 1nkw_0
**CGU**	DII(C1257.G583). DI(U448)<br>*NASSAM hit in 2j01_A*	(C1361.G640). (U454) 1ffk_0<br>(C1257.G583). (U448) 2awb_B<br>(C1270.G592). (U460) 1nkw_0
**GGU**	DV (G2092^{t}.G2093^{t}.U2652^{t})<br>*Intra-helix interaction (helix G.U to G2092 on bulge).*	(A2051.A2052.U2617) 2awb_B<br>(A2051.G2052.C2617) 2j01_A<br>(A2034.G2035.C2596) 1nkw_0
**GUU**	DV (U2495^{t}.G2525^{t}.U2527^{t})<br>*Intra-helix interaction.*	(U2460.G2490.U2492) 2awb_B / 2j01_A<br>(U2439.G2469.U2471) 1nkw_0
	DII (G739.U740.U757)<br>*NASSAM hit in 2j01_A*	(U831.U832.U850) 1ffk_0<br>(A739.C740.G757) 2awb_B<br>(G752.U753.U770) 1nkw_0

1fjg_A 16S rRNA from *T. thermophilus*
5D=5' domain, CD=central domain, 3MAD=3' major domain
Base positions which were observed as conserved when aligned with 20 other prokaryotic 16S sequences are underlined.

Triple	Triple location	Structural interaction / Observation
**AAA**	5D (A411.A414.A430)	Interactions within internal loop.
**AAG**	3MAD (A978.G1316.A1360)	(Multi) Loop – (multi) loop – (multi) loop (G1316) interactions.
**ACG**	5D (A160.G347.C342)	Helix G.C (near hairpin loop) to A in hairpin loop.
	CD (A608). 5D (G292.C308)	Helix G.C to A in internal loop.
	5D (C54.A55.G357)	Helix G.C to A on bulge.
**ACU**	3MAD (A1055.C1200.U1205)	Intra helix interaction (A on bulge, C on bulge, U on helix).
	5D (C372.U375.A389)	Interactions within internal loop.
**AGG**	3MAD (G1160.A1176.G1182)	Helix A.G to multi loop.
	5D (G64.G68.A101)	Helix A.G to G64 on bulge loop.
**AGU**	5D (A55.G357.U368)	Inter helix interactions (G,A bulge, same stem) to U bulge (another helix).
	3MAD (U1315.A1319.G1361)	A and U in hairpin loop to G in hairpin loop.
**AUU**	5D (U13.U20). CD (A915)	U.U in hairpin loop to A in multi loop.
**CGG**	3MAD (C1158.G1177.G1181)	Helix G.C to G1181 on strand.
	5D (G251.G254.C272)	Helix GC to G251 in bulge loop.
	3MAD (G1053.G1057.C1203)	Intra helix interaction (helix G.C to G1053).

Table S3. Geometric families according to the classification system of Leontis and Westhof [23] for triples in the 23S rRNA subunit of *H. marismortui* (H = Hoogsteen edge, S = Sugar edge, WC = Watson-Crick edge).

Triple	Leontis and Westhof Geometry	
	**1ffk_0 numbering and geometry of triple**	**Geometric family**
**AAC**	Tran3s A509 WC / A485 WC – Trans A509 H / C505 WC	Trans H / WC – Trans WC / WC
**AAG**	Cis G452 H / A460 WC – Cis A460 H / A455 WC	Cis H / WC – Cis H / WC
**AAU**	Cis 2095 WC / U2612 WC – Cis 2095 H / A2612 WC	Cis H / WC – Cis WC / WC
	Cis 2321 WC / U2378 WC – Trans A2321 S / A2380 H	Cis WC / WC – Trans S / H
	Trans A485 WC / A509 WC – Trans A485 H / U481 WC	Trans H / WC – Trans WC / WC
	Trans A2369 WC / A2356 WC – Trans A2369 H / U2330 WC	Trans H / WC – Trans WC / WC
	Trans A1682 H / A1414 WC – Trans A1682 WC / U1696 WC	Trans H / WC – Trans WC / WC
**ACC**	Cis A2485 H / C2536 WC – Cis A2485 WC / C2104 WC	Cis H / WC – Cis WC / WC
	Cis A1005 H / C959 WC – Cis A1005 WC / C963 WC	Cis H / WC – Cis WC / WC
**ACG**	Cis G2731 S / A1811 WC – Cis G2731 WC / C2751 WC	Cis S / WC – Cis WC / WC
	Cis G2449 S / A1921 WC – Cis G2449 WC / C418 WC	Cis S / WC – Cis WC / WC
	Cis G416 S / A198 WC – Cis G416 WC / C424 WC	Cis S / WC – Cis WC / WC
	Cis G2657 S / A2841 WC – Cis G2657 WC / C2087 WC	Cis S / WC – Cis WC / WC
	Cis G871 S / A776 WC – Cis G871 WC / C879 WC	Cis S / WC – Cis WC / WC
	Cis G958 S / A961 WC – Cis G958 WC / C1008 WC	Cis S / WC – Cis WC / WC
	Cis G2033 H / A1742 H – Cis G2033 WC / C2037 S – Trans A1742 WC / C2037 WC	Cis H / H – Cis WC / S – Trans WC / WC
**ACU**	Cis A1942 WC / U1897 WC – Trans A1942 H / C1894 WC	Cis WC / WC – Trans H / WC
	Cis A926 WC / U917 WC – Trans A926 H / C1043 WC	Cis WC / WC – Trans H / WC
	Cis A1437 WC / U1429 WC – Trans A1437 H / C1426 WC	Cis WC / WC – Trans H / WC
**AGU**	Cis G924 S / A166 WC – Trans G924 WC / U919 WC	Cis S / WC – Trans WC / WC
**AUU**	Trans A2054 H / U1371 WC – Trans A2054 WC / U2648 WC	Trans H / WC – Trans WC / WC
	Cis A339 WC / U308 WC – Trans A339 H / U335 WC	Cis WC / WC – Trans H / WC
	Cis A2118 WC / U2276 WC – Cis A2118 H / U2116 WC	Cis WC / WC – Trans H / WC
	Cis A2470 WC / U2115 WC – Cis A2470 H / U2277 WC	Cis WC / WC – Trans H / WC
	Cis U2791 WC / A2793 H – Trans U2791 H / U2781 WC	Cis WC / H – Trans H / WC
	Cis A2074 WC / U619 WC – Trans A2074 H / U2069 WC	Cis WC / WC – Trans H / WC
**CGG**	Cis G868 WC / C884 WC – Trans G868 H / G775 WC	Cis WC / WC – Trans H / WC
	Cis G702 WC / C726 WC – Cis G702 H / G744 WC	Cis H / WC – Cis WC / WC
	Cis G84 WC / C62 WC – Cis G84 H / G56 WC	Cis H / WC – Cis WC / WC
	Cis C171 WC / G164 WC – Trans C171 S / G221 WC	
	Cis G2617 WC / C2541 WC – Cis G2617 S / G2588 S – Trans G2588 S / C2542 S	Cis S / S – Cis WC / WC – Trans S / S
	Cis C2405 WC / G2383 WC – Trans C2405 H / C2419 WC	Cis WC / WC – Trans H / WC
	Cis G2700 WC / C2695 WC – Trans G2700 S / G2567 S	Cis WC / WC – Trans S / S
**CGU**	Cis G640 WC / C1361 WC – Trans G640 S / U454 WC	Cis WC / WC – Trans S / WC
**GGU**	Cis G2093 WC / U2652 WC – Cis G2093 H / G2092 S – Cis G2092 WC / U2652 WC	Cis H / S – Cis WC / WC – Cis WC / WC
**GUU**	Cis 2495 WC / U2527 WC – Cis U2527 H / G2525 H – Cis G2525 WC / U2495 WC	Cis H / H – Cis WC / WC – Cis WC / WC

References

1. Burkard, M.E., Turner, D.H. and Tinoco Jr., I. (1999) In Gesteland, R. F., Cech, T. R. and Atkins, J. F. (eds.), *The RNA World - Second Edition*. Cold Spring Harbor Laboratory Press, pp. 675-680.
2. Nagaswamy, U., Larios-Sanz, M., Hury, J., Collins, S., Zhang, Z.D., Zhao, Q. and Fox, G.E. (2002) NCIR: a database of non-canonical interactions in known RNA structures. *Nucleic Acids Res.*, 30, 395-397.
3. Walberer, B.J., Cheng, A.C. and Frankel, A.D. (2003) Structural diversity and isomorphism of hydrogen-bonded base interactions in nucleic acids. *J. Mol. Biol.*, 327, 767-780.
4. McDonald, I.K. and Thornton, J.M. (1994) Satisfying Hydrogen-Bonding Potential in Proteins. *J. Mol. Biol.*, 238, 777-793.
5. Ravelli, R.B.G., Leiros, H.K.S., Pan, B.C., Caffrey, M. and McSweeney, S. (2003) Specific radiation damage can be used to solve macromolecular crystal structures. *Structure*, 11, 217-224.
6. Ban, N., Nissen, P., Hansen, J., Moore, P.B. and Steitz, T.A. (2000) The complete atomic structure of the large ribosomal subunit at 2.4 Angstrom resolution. *Science*, 289, 905-920.
7. Nissen, P., Hansen, J., Ban, N., Moore, P.B. and Steitz, T.A. (2000) The structural basis of ribosome activity in peptide bond synthesis. *Science*, 289, 920-930.
